# Understanding the *Cryptosporidium* species and their challenges to animal health and livestock species for informed development of new, specific treatment strategies

**DOI:** 10.3389/fpara.2024.1448076

**Published:** 2024-08-06

**Authors:** Hannah Rideout, Alasdair J. C. Cook, Anthony D. Whetton

**Affiliations:** ^1^ Veterinary Health Innovation Engine, University of Surrey, Guildford, United Kingdom; ^2^ School of Veterinary Medicine, Faculty of Health and Medical Sciences, University of Surrey, Guildford, United Kingdom; ^3^ School of Biosciences, Faculty of Health and Medical Sciences, University of Surrey, Guildford, United Kingdom

**Keywords:** *Cryptosporidium*, treatment strategies, livestock, animal health, parasite challenges

## Abstract

*Cryptosporidium* species are parasitic organisms of vertebrates with a worldwide distribution. They have an important impact globally upon human and animal health, and livestock productivity. The life cycle of these species is complex and difficult to disrupt to improve human health, animal health, food security and economic growth. This may contribute to the fact that no new treatment strategy has been widely accepted or applied in livestock for years. Here we consider the natural history of these parasites, their biochemistry and economic impact. Using recent developments in understanding these parasites we then consider viable and affordable approaches to enhancing control of their effects on livestock. These are based on advances in drug discovery, omics research and artificial intelligence applications to human and veterinary medicine that indicate putative new therapeutic approaches.

## 1 *Cryptosporidium* species

**Figure 1:**
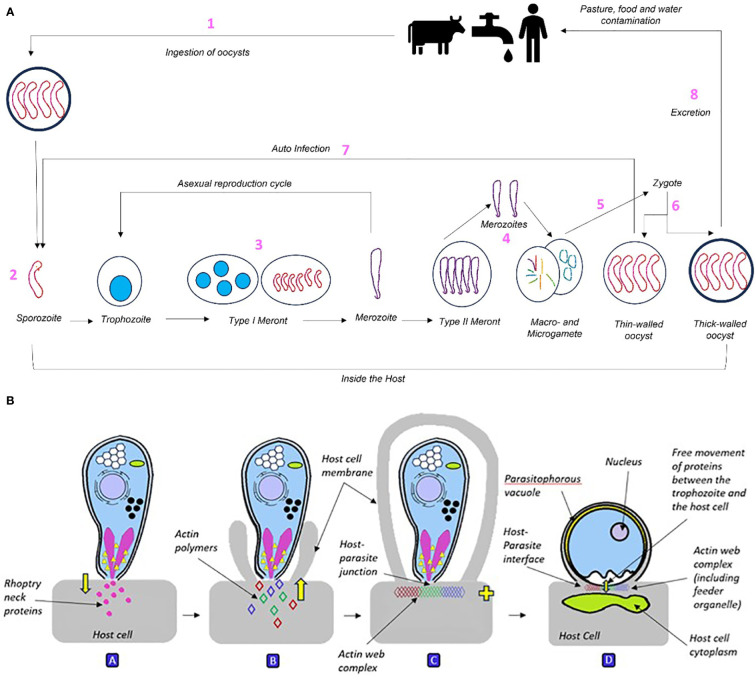
**(A)** The *Cryptosporidium* life cycle broken down into 8 component stages: 1) Pathogen is ingested and begins forming a feeder organelle responsible for nutritional intake. 2) Pathogen attaches to cell surface, provoking cell to embrace rather than active invasion. 3) Once inside the host cell, the sporozoite divides asexually three times into a type I meront, containing eight merozoites. Merozoites then either begin asexual development cycle to produce more type I meronts or differentiate into type II meronts. 4) Type II meronts contain four merozoites that develop by asexual division, they are divided into micro- and macro-gametes. 5) Mature micro-gametes fertilise macro-gametes and leave the cell, producing zygotes. 6) Zygotes go through sporogony (meiosis) to form sporulated oocysts. 7) Thin-walled oocysts (20%) lead to endogenous autoinfection. 8) Thick-walled oocysts (80%) represent exogenous stage and are excreted. The global estimated load of *Cryptosporidium* in livestock manure is a huge 3.2 x10^23^ oocysts per year, predominantly sourced from cattle (Vermeulen et al., 2017). **(B)** *Cryptosporidium* sporozoite interacting with the host cell before forming into an intracellular trophozoite. A: The sporozoite attaches to the host cell via apical contact, inducing discharge of rhoptry neck (binding) and bulb (pathogenic) proteins into the host cell. B: This causes the host cell membrane to engulf the sporozoite, attracting host actin polymers towards the parasite-host interface. C: As the host membrane fully engulfs the sporozoite, host and parasite actin polymers bind into a plaque-like multiprotein web complex of unknown composition at the host-parasite junction. D: Structure of the *Cryptosporidium* trophozoite. As the host cell cytoskeleton engulfs the sporozoite, this causes it to curve into a c-shape, relax into a straight line then contract into a rounded trophozoite (above). The trophozoite’s niche is that it is intracellular within the host membrane yet extra-cytoplasmic due to the formation of the parasitophorous vacuole, and partial separation from the host cell by the actin web. As the trophozoite is the growth stage of the parasite, it relies heavily on the host cell for metabolites and nutrients. This is achieved via the formation of the feeder organelle at the host-parasite interface during fusion with the host cell.

*Cryptosporidium* are protozoan pathogens that result in the enteric disease cryptosporidiosis (Helmy and Hafez, 2022). Previously they were classified as coccidia and considered to be intracellular parasites. However, recent research states they are members of a new class named cryptogregaria. In evolutionary terms this lies between coccidia and gregarine (Cavalier-Smith, 2014). Features that set them apart from coccidia include intracellular and extra-cytoplasmic localisation, feeder organ formation, presence of morphological oocysts, smaller oocysts, absence of sporocysts or micropyles, and, importantly, resistance to all available anti-coccidial drugs (Hijjawi et al., 2002; Smith and Corcoran, 2004; Helmy and Hafez, 2022). The re-classification has not been challenged since its publication meaning *Cryptosporidium* is now officially gregarine (Ryan et al., 2016). Gregarines are single-celled apicomplexan parasites that primarily infect the intestines of invertebrates and lower vertebrates (Leander et al., 2003a; Leander et al., 2003b); Barta and Thompson, 2006; Leander, 2007; Valigurová et al., 2007). The transmission of gregarines to new hosts usually takes place by water borne or land-based ingestion of oocysts, see **Figure 1A** (Thompson et al., 2016). This has recently been seen in an outbreak in Southern England likely due to bovine faeces contamination of water supplies (BBC, 2024).

There are over 40 recognised *Cryptosporidium* species, affecting a variety of hosts, with ten posing zoonotic risk, eight affecting livestock, and three affecting poultry (Fayer, 2010; Chalmers and Katzer, 2013; Thomson, 2016; Feng et al., 2018). The most frequently identified species globally is *C. parvum*, which almost exclusively presents clinically in neonates as watery diarrhoea, appetite loss, abdominal pain, and in extreme cases, death (Thomson et al., 2017). Infection with *C. parvum* is the most common cause of neonatal calf diarrhoea (NCD) worldwide, also causing significant clinical disease in humans, lambs, and goats (Thomson et al., 2017). Diarrhoea may be malabsorptive, caused by villi blunting and loss of enterocytes, or secretory, when intestinal secretion increases (Thomson, 2016). Other species such as *C. ryanae* and *C. bovis* are shed by parasitised hosts, although not associated with clinical disease (Fayer et al., 2008). Some species multiply in the respiratory tissue such as *C. baileyi*, causing respiratory symptoms and disease (Helmy and Hafez, 2022). *C. parvum* is the only species proven to have zoonotic effects (**Table 1**) and some human outbreaks have been attributed to waterborne infection from cattle. *C. hominis* (formerly *C. parvum* genotype I), has been shown to be specific to humans. Although *Cryptosporidium* spp. are generally host specific, species including *C. suis, C. baileyi* and *C. bovis* have occasionally been detected in other animals and humans (Helmy and Hafez, 2022).

**Table 1:** Most pathogenic *Cryptosporidium* species, their host and location.

*Cryptosporidium* spp.	Main Hosts	Location
*C. parvum*	Humans, Cattle, Sheep, Pigs	Small Intestine
*C. bovis*	Cattle	Small Intestine
*C. ryanae*	Cattle	Small Intestine
*C. andersoni*	Cattle	Abomasum
*C. ubiquitum*	Sheep	Small Intestine
*C. xiaoi*	Sheep	Small Intestine
*C. suis*	Pigs	Small Intestine
*C. baileyi*	Chickens	Cloaca, bursa, trachea
*C. meleagridis*	Chickens	Small Intestine

Collated from (Sréter and Varga, 2000) and (Helmy and Hafez, 2022).

## 2 Market overview

**Figure 2:**
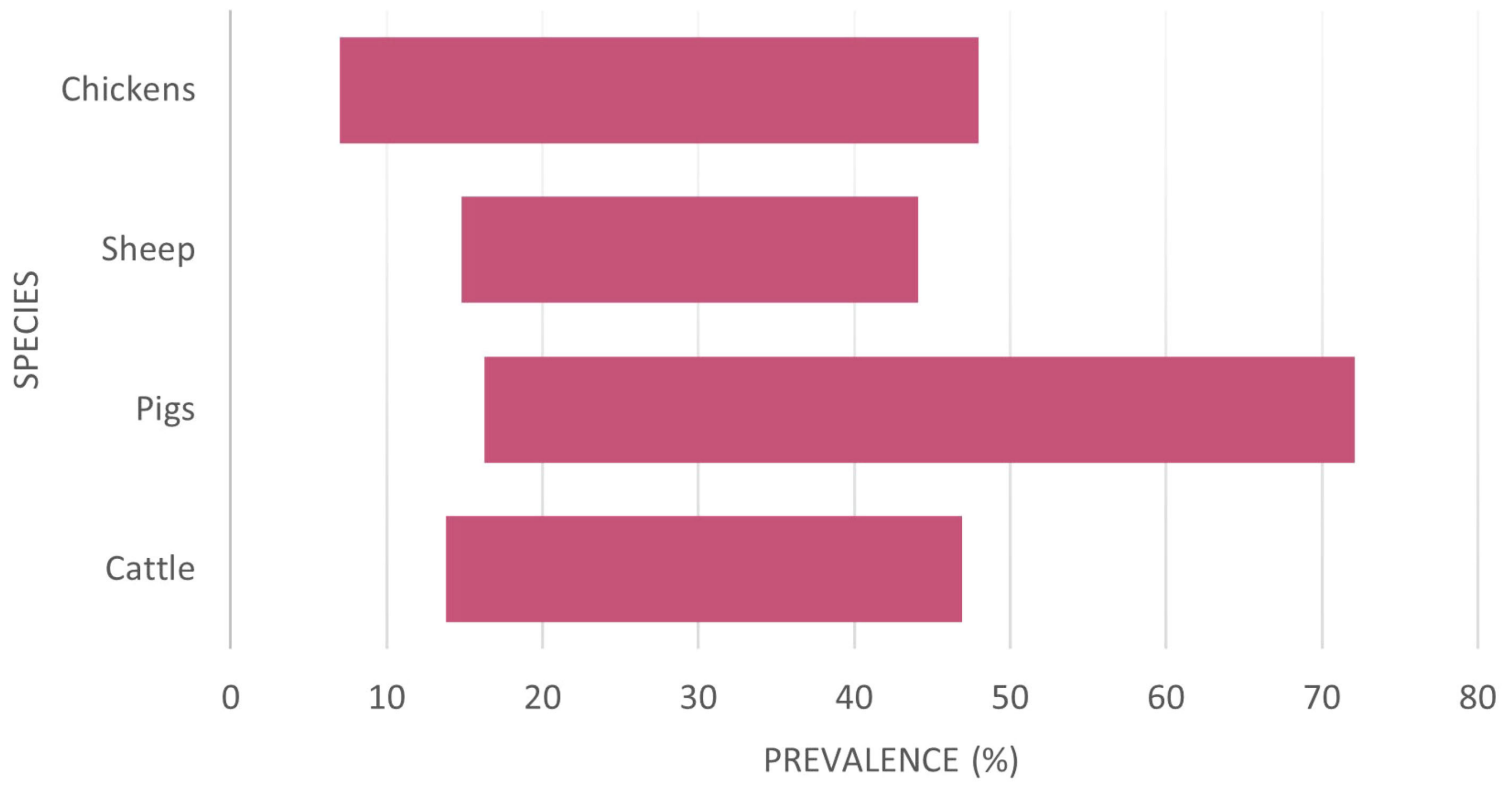
The variation of reported *Cryptosporidium* prevalence per species considered from a global perspective (raw data in **Appendix 1**). There is apparently no country with significant livestock populations free from *Cryptosporidium*.

There is a need to consider the economic drivers that require advances in understanding and treatments for *Cryptosporidium* infections. *Cryptosporidium* has a major agri-economic effect on cattle. There are over 142 million dairy cattle worldwide, the highest number being in India (61 million), followed by the EU (20 million) and the US (9.5 million). The average volume of milk consumed per person worldwide is 30.3kg/year, expected to grow annually by 6.87% (Shahbandeh, 2023), demonstrating the growing need for new approaches to treat this disease and maintain food security. *Cryptosporidium* prevalence varies between hosts, geographical area, and diagnostic test used (**Table 1**; **Figure 2**).

The critical facet by which *Cryptosporidium* affects the livestock economy is neonatal calf diarrhoea or NCD, the most common cause of death in dairy calves, that also causes high morbidity, growth retardation and other serious long-term consequences (Brunauer et al., 2021). There are several viral, bacterial, and parasitic causative agents of NCD; but one of the most frequently identified at <30 days old is *Cryptosporidium* (57%) (Thomson et al., 2017; Brunauer et al., 2021). Whilst the economic case is made most clearly in the literature for cattle, other livestock also suffer from *Cryptosporidium* infections. Thus, the cost across all livestock species has a profound effect on agriculture. Notably, the prevalence of *Cryptosporidium* infections in pigs globally is reported as generally being high, especially in in post-weaned pigs. However, pre-weaned pigs and finishing pigs are majorly impacted by the disease in some regions such as China and Vietnam (Nguyen et al., 2012; Wang et al., 2022; Chen et al., 2023).

In the US, to raise a beef calf to 24 months costs approximately £1,763 ($2,260). Shaw et al. (2020) noted that a loss of 34kg/head due to cryptosporidiosis would equate to a loss of £128.18/head, not accounting for extra feed and husbandry costs, diagnostics, vet fees or treatment. Therefore, measures to prevent and control shedding in livestock will benefit both human and livestock health and welfare significantly, whilst also increasing production efficiency (Innes et al., 2020). Economic losses associated with *Cryptosporidium* are variously linked with mortality, decreased growth rates, medicine requirement, veterinary care costs, and increased labour (de Graaf et al., 1999). Thus, improving the health and welfare of dairy calves is imperative to sustaining the industry and decreasing methane (greenhouse gas emitted from cattle) (Roblin et al., 2023). In economically less developed countries, *Cryptosporidium* is a serious issue due to the limited resources and diagnostics availability, lower hygiene, and poorer livestock practices (Ahmed and Karanis, 2020; Fresán et al., 2021). In other species, mortality rates due to cryptosporidiosis in lambs equate to £40–100 million per year in the UK (Cutress and Rees, 2022). Similarly, a decreased growth rate in lambs was reported to equate to a loss of approximately £7/head (Sweeny et al., 2011; Agriculture and Horticulture Development Board, 2023). Economic losses concerning pig and poultry production are currently undocumented, though collectively, the data available demonstrates the importance of *Cryptosporidium* parasites in affecting agricultural production in these species too.

The economic case for improved prophylactic treatment measures can be made, but any form of treatment, prophylactic or otherwise, would have to consider the health-economic case based on the incidence of the disease. The only licensed treatment for *Cryptosporidium* in calves is halofuginone lactate (HL) (Jain et al., 2015), licensed in regions such as the UK and EU, though not in the US. A metareview of the literature recently argued that the evidence is growing for the prophylactic use of this drug (Brainard et al., 2021). But HL cannot be administered to calves that are diarrhoeic for >24 hours when they are weaker and dehydrated (European Medicines Agency, 2021). Thus, its use as a therapeutic agent is limited given *Cryptosporidium* oocysts survive a range of different cleaning agents, temperatures, and conditions, including disinfectants. Therefore, we need to find new, cost-effective treatments whilst halofuginone can be used prophylactically.

## 3 Species specific consideration of the effects of *Cryptosporidium* infection

### 3.1 Cattle

There are over 18 *Cryptosporidium* species that affect cattle; the main four documented are *C. parvum, C. bovis, C. ryanae* and *C. andersoni* (Thomson et al., 2017). These species have been shown to affect cattle on an age-related scale; *C. parvum* is predominantly found in neonatal calves, *C. bovis* and *C. ryanae* are found in post-weaned calves, and *C. andersoni* is found in adults (Díaz et al., 2021). The minimum infectious dose for clinical presentation in neonatal calves is an amazingly small number of 17 oocysts, with *C. parvum* being detected as early as day-old calves (Zambriski et al., 2013; Thomson et al., 2019). This number shows the challenge in prevention, prophylaxis, and treatment, as one primary case can rapidly lead to an exponential incidence in infected animals. Stocking density is a significant risk factor in the prevalence of *C. parvum* in dairy calves. The prevention and control of this zoonosis in dairy calves should receive greater attention, especially in regions with a high degree of intensive dairy farming. Co-infection with other pathogens and/or non-pathogenic diarrhoeal causes can exacerbate symptoms and is one of the major causes of mortality in calves (Björkman et al., 2018). The main sources of infection are shedding of oocysts by infected neighbouring pens, and contamination of bedding, water supply, and udders/teats of dams (Helmy and Hafez, 2022).

Cryptosporidiosis influences calf productivity via costs of mortality, diagnosis, treatment, and extra husbandry to reach market weight (Olson et al., 2004). *Cryptosporidium* infections have been shown to directly correlate to a lower live weight gain and poorer productivity (Abreu et al., 2019). Shaw et al. (2020) found that any weight lost during infection in neonatal calves is not put back on in the subsequent six months, indicating that the impact of cryptosporidiosis on the cattle industry may be greater than originally thought.

### 3.2 Chickens

Unlike mammals, cryptosporidiosis in poultry manifests in two forms, respiratory and intestinal (Sréter and Varga, 2000). *C. baileyi* causes the respiratory form, affecting the nasal turbinates, nasopharynx, sinuses, larynx, trachea, lungs, air sacs and conjunctiva (Sréter and Varga, 2000). Respiratory infection clinically presents as depression, lethargy, anorexia, un-thriftiness, coughing, sneezing, dyspnoea, and conjunctivitis (Sréter and Varga, 2000). *C. meleagridis* causes the intestinal form resulting in diarrhoea, enteritis, and dehydration (Nakamura and Meireles, 2015). *C. parvum* is rarely reported to be the cause of cryptosporidiosis in chickens, though a more recent study challenges this (Berrilli et al., 2012; Helmy et al., 2017). For reasons unknown, prevalence of cryptosporidiosis in chickens has been found to be higher in layers than broilers (Helmy et al., 2017).

### 3.3 Pigs

The most common species found in pigs are *C. parvum, C. suis* and *C. scrofarum* (Zahedi and Ryan, 2020). Unlike ruminants, pigs are largely asymptomatic towards *Cryptosporidium* infections, unless the animal is immunocompromised or under extreme stress (Ramirez et al., 2004). Although, nursing piglets (under 3 weeks) have been reported to have serious clinical presentations (Rotkiewicz et al., 2001).

### 3.4 Small ruminants

*C. parvum, C. ubiquitum*, and *C. xiaoi* are the most frequently detected species in small ruminants, with *C. andersoni, C. bovis, C. ryanae, C. hominis, C. fayeri, C. baileyi*, and *C. suis* all identified sporadically (Santin, 2020). Clinical presentation results in acute diarrhoea between 5–20 days, causing reduced growth and productivity in lambs and kids (Paraud and Chartier, 2012; Jacobson et al., 2016). Oocyst shedding has been associated with lower carcass weight in both symptomatic and asymptomatic sheep (Jacobson et al., 2016).

### 3.5 Zoonotic risk

Zoonotic transmission of *C. parvum* can occur via contaminated drinking water or food, crops fertilised with contaminated manure, direct contact with infected humans or animals, and anal sexual contact (Dillingham et al., 2002; McKerr et al., 2015). *Cryptosporidium* is the biggest pathogenic threat to the water industry, accounting for most outbreaks of infectious intestinal disease between 2004–2010 (Chalmers, 2012) (Karanis, 2018). In the US, exposure to contaminated swimming pools and water parks resulted in 35% of the reported cryptosporidiosis outbreaks between 2009–2017 (Gharpure et al., 2019). Despite this, little is understood about how oocysts reach the water source. The recent outbreak of cryptosporidiosis in Devon, UK, highlights the impact that the parasite can have on public health, with residents experiencing symptoms up to 10 days before acknowledgment and advice from the water company where infection was likely from bovine faeces (BBC, 2024).

Analysis of meta-data throughout a range of studies (Dong et al., 2020) found global human prevalence of *Cryptosporidium* to be 7.6% in 2020; the highest prevalence was found in Mexico (70%) followed by Nigeria (34%), Bangladesh (42%) and Republic of Korea (8%). Prevalence has been found to be higher in people from low-and-middle-income countries (LMIC), children under five, and those living in rural areas (Dong et al., 2020). For example, people in rural areas of China have a significantly higher prevalence of Cryptosporidiosis infections (1.8–12.9%) than those in urban areas (0–3.7%), likely due to poor sanitation (Liu et al., 2020).

The incubation period of *Cryptosporidium* in humans is 5–21 days, preceding a self-limiting diarrhoea lasting 3–12 days. Clinical signs include diarrhoea, vomiting, abdominal pain, nausea, fatigue and on some occasions, respiratory symptoms such as coughing and sneezing due to inhalation of oocysts from contaminated air (Sponseller et al., 2014; Ahmed and Karanis, 2020). In many countries, cryptosporidiosis is a major concern due to the increasing number of immunocompromised adults and children (Pumipuntu and Piratae, 2018). For example, in regions of Africa where HIV/AIDS outbreaks are still common, there is a much higher prevalence of cryptosporidiosis (Squire and Ryan, 2017). The incidence of cryptosporidiosis is also increasing with climate change as heavier rainfall and flooding increase the likelihood of pathogenic water contamination (Gertler et al., 2015). Human-infective pathogens are subject to more intensive experimental research than those affecting cattle. Excessive anticoccidial residues in animal-derived food (i.e., meat, eggs, etc.) have been associated with toxicological effects such as teratogenicity, hepatotoxicity, or neurotoxicity (Tuck et al., 2016). Additionally, inappropriate usage or excessive administrations can cause harmful side effects in humans such as resistance, carcinogenicity, organ dysfunction and other adverse effects (Zhou et al., 2018).

## 4 Diagnosis and treatment

### 4.1 Diagnostic procedures

#### 4.1.1 Diagnostic tests

Conventionally, microscopic identification is used to confirm presence of oocysts in animal stool samples, including various staining techniques (Ziehl-Neelsen, safranin-methylene blue etc.). Additionally, faecal concentration techniques are performed by sedimentation using formalin-ethyl acetate or floatation using zinc sulphate (ZnSO_4_) or salt (NaCl) (Pumipuntu and Piratae, 2018). Microscopic techniques are time consuming and laborious, with misdiagnosis frequent due to insufficient knowledge of oocyst morphology. Therefore, immunological techniques have now been developed such as protein measurement-based Enzyme Linked Immunosorbent Assays (ELISAs), immunofluorescence to detect oocyst cell wall antigens using specific fluorescent antibodies, and dipstick-like tests (Fayer et al., 2000). Antigen detection kits mean that many samples can be processed easily and quickly, with 98–100% specificity (Ghoshal et al., 2018). Immunochromatography (ICT) or enzyme immunoassay (EIA) tests are available for presence of *Cryptosporidium* alone, or in combination with others parasite tests, such as for *Giardia*. A recent and comprehensive summary of these tests has been published (Aboelsoued and Megeed, 2022). Although these approaches are more sensitive and specific, reducing labour, time and cost, there are no antibodies to differentiate between *Cryptosporidium* species reliably (Pumipuntu and Piratae, 2018). Molecular methods (e.g., polymerase chain reaction – PCR) are increasingly used in reference diagnostic labs, since they can be used to identify *Cryptosporidium* at the species level and thus convey sensitivity and specificity. Such an assay was first published in 1995 (Johnson et al., 1995).

#### 4.1.2 Molecular diagnostic techniques

PCR tests are repeatable, automated, sensitive, and specific techniques, giving them significant advantages over other techniques. However, the quality and purity of DNA in the sample affects the sensitivity of the test (Checkley et al., 2015). The issue for *Cryptosporidium* diagnosis and monitoring are the speed with which an assay can be performed, and the cost of such assays, these two facets are linked. Rapid PCR reduces the time required for assays to be performed, but testing costs are higher (Fistera et al., 2023). Nonetheless, a rapid assay for several *Cryptosporidium* species has been developed (Crannell et al., 2014). The technique employed, Recombinase Polymerase Amplification (RPA), is an isothermal nucleic acid amplification technique that has been widely used to detect different kinds of pathogens (Piepenburg et al., 2006). RPA can achieve exponential amplification of the target fragment in less than 30 minutes. This RPA assays was described as easier and faster to carry the detection results than existing methods, plus suitable for point-of-care detection and valuable in prevention and control of infections. The key question remains the economic cost inclusive of the assay, staff time required, and frequency of use requirement (Liu et al., 2023).

Loop-mediated isothermal amplification (LAMP) is an isothermal amplification technique that discard complex thermal cycling instruments and amplify the target fragment at a constant temperature. LAMP is said to be low-cost, accurate and highly sensitive but it is argued that high rates of false positive results are an issue for clinical decision making, though there are means suggested to overcome this issue (de Oliveira Coelho et al., 2021; Alhamid et al., 2023). LAMP can be used to detect *Cryptosporidium* with the ability to differentiate between six regions of the target gene (Plutzer et al., 2010). LAMP has been used to detect a gastrointestinal nematode in ovine populations and bovine respiratory diseases (Khangembam et al., 2021; Pascual-Garrigos et al., 2021). Thus, it has been suggested as a point of care test. LAMP is faster than PCR due to it not requiring an incubator or thermocycler, also being superior to nested PCR at detecting low numbers of *Cryptosporidium* in apparently healthy animals (Aboelsoued and Megeed, 2022). However, another field-based approach has been proposed; a sensitive assay for *Cryptosporidium* detection using a portable platform. This method is more sensitive, selective, and PCR-free, detecting *Cryptosporidium* RNA using oligonucleotide modified gold nanoparticles (AuNPs). The need for laboratory use was obviated by undertaking assays on a chip in a three-dimensional printed holder assembly. A smartphone camera was used to capture an image of the colour change for quantitative analysis. This approach may fill many of the needs of the livestock industry if cost effective (Luka et al., 2021).

#### 4.1.3 Postmortem findings

The final and postmortem method is pathology based where findings for *Cryptosporidium* infection include generalised enterocolitis, inclusive of curdled milk in abomasum, petechiae in abomasal mucosa, intestinal serosa, and mesentery, enlarged lymph nodes, and congested, ballooned small intestines (Bharti et al., 2020). Similarly, histopathology findings are ileitis (characterised by villous atrophy and necrosis), epithelial erosion, leukocyte infiltration into lamina propria, and mucosal haemorrhage (Gamsjäger et al., 2023).

### 4.2 Treatments and control

#### 4.2.1 Best husbandry practices

Transmission of C*ryptosporidium* occurs via a faecal-oral route; thus, water sources easily contaminated with oocysts, lead to outbreaks of cryptosporidiosis (Thomson, 2016). Oocyst shedding can be high from infected hosts, such that faecal material can lead to the parasite passing quickly to animals or humans. Infected calves have been shown to shed around 1x10^4^-10^8^ oocysts/gram faeces 4–9 days after initial infection, for around 6–14 days (Fayer et al., 1998; Faubert and Litvinsky, 2000; Zambriski et al., 2013). Infected animals can vary in the degree of oocyst shedding based on the infectious dose (Zambriski et al., 2013).

The life cycle of *Cryptosporidium* is complex. It consists of sexual and asexual reproductive stages (Khan and Witola, 2023). Tandel et al. (2019) suggest direct development of gametes from type I meronts may occur, which has only been reported in *Cryptosporidium* species (**Figure 1B**). The data on life cycles indicate that good husbandry practice, clean water supplies and surveillance can all help deal with infection and infection rates. Affected animals should be quarantined for at least 1 week after the diarrhoea subsides to avoid subsequent oocyst shedding from contaminating the environment causing further transmission (Innes et al., 2020). Efficient biosecurity and disinfection of shared spaces (i.e., calf pens) is the best control strategy, though this is easier said than done. Clean, dry housing with raised feed and water troughs minimises chances of contamination and/or exposure to parasites from the environment (Innes et al., 2020). *Cryptosporidium* spp. have developed resistance to most chemical disinfectants (Fayer et al., 2008). However, Björkman et al. (2018) found that disinfection of the environment with hydrated lime, whilst not a cure, is an effective way to reduce onset and severity of cryptosporidiosis in calves. Other interventions making the oocyst more sensitive to disinfectants would also be beneficial (see below). Additionally, steam cleaning of enclosures between animals has been shown to be effective, as oocysts become inactivated above 60°C (Robertson et al., 1992). *C. parvum* oocysts can survive temperatures of -20°C, meaning they may be able to survive cryoprotectants. However, UV light exposure has been shown to render oocysts non-infectious (Sivaganesan and Sivaganesan, 2005).

#### 4.2.2 Therapeutic approaches

As immunocompromised, neonatal, and malnourished patients are the most vulnerable to the pathogen, they are most in need of therapeutic treatment (Khan and Witola, 2023). Ensuring calves receive an adequate supply of colostrum to boost their immune system in their most naïve state is therefore essential for controlling the clinical disease (Meganck et al., 2014). Oral rehydration therapies are a therapeutic treatment option for cryptosporidiosis due to the diarrhoea and lethargy associated with the clinical disease. But the discovery of new drugs to treat livestock for cryptosporidiosis has had considerable investment recently, with several classes of compounds in various research stages (Innes et al., 2020). These include studies on both *Cryptosporidium*, and related parasites to identify new compounds (Lendner et al., 2015; Lee et al., 2018; Buckner et al., 2019; Lunde et al., 2019). Additionally, given that cryptosporidiosis is zoonotic, advancements in human drug development may be transferrable to livestock in the future. Several drugs have been tested in humans including macrolides, rifamycin, letrazuril, paromomycin, nitazoxanide, clofazimine. With livestock production moving further towards sustainability and reducing environmental impact, methods of tackling the disease with biodegradable drugs are increasingly important.

Despite investment in discovery, deployment of new treatments has moved at an extremely slow pace. There are only two treatment options available for calves: HL and paromomycin (**Table 2**). Both are used at the onset of clinical signs to reduce shedding and severity of diarrhoea (Grinberg et al., 2002; Trotz-Williams et al., 2011). HL, whilst registered for use in several countries (not the US), has a substantial market price, narrow therapeutic index and displays toxicity at twice the recommended dose (Health Products Regulatory Authority, 2021). Almawly et al. (2013) also found that the anti-cryptosporidial activity of HL is not preserved during co-infection with Rotavirus and S*. typhimurium* in calves; this is said to be due to diarrhoea, caused by these pathogens, shortening gut transit time of HL, abolishing its effects.

**Table 2:** List of currently licensed drugs for cryptosporidiosis in production animals.

Licensed Drugs for Cryptosporidiosis									
Drug Name	Manufacturer	Route of administration	Cost (£/ml)	Dosage	Ingredients		Withdrawal period (days)	Legal category	Licensed for
					Actives	mg/ml			
HALOCUR	MSD	Oral	0.13	35–45kg: 8ml/day for 7 days; 46–60kg: 12ml/day for 7 days; For other weights: 2ml/10kg BW	Halofuginone	0.5	13	POM-V	Calves
Gabbrovet Multi	Ceva	Oral	0.49	150mg/kg/day for 5 days	Paromomycin	140	Cattle: 110; Pigs: 3	POM-V	Cattle, Pig
Parofor crypto	Huvepharma	Oral	0.28	2.5ml/10kg BW/day for 7 days	Paromomycin	140	62	POM-V	Non-ruminant calf
Kriptazen	Virbac	Oral	0.23	2ml/10kg BW/day for 7 days	Halofuginone	0.5	13	POM-V	Calves
STENOROL CRYPTO	Huvepharma	Oral	Unknown	2ml/10kg BW/day for 7 days	Halofuginone	0.5	13	POM-V	Cattle
Halofusol	Bimeda	Oral	0.27	35–45kg: 8ml/day for 7 days; 46–60kg: 12ml/day for 7 days; For other weights: 4ml/20kg BW	Halofuginone	0.5	13	POM-V	Cattle
Parofor Crypto Sheep and goats	Huvepharma	Oral	0.19	0.25ml/kg/day for 7 days	Paromomycin	140	24	POM-V	Non-ruminant lambs and kids

Triazine-based anti-protozoal drugs are used in the treatment of apicomplexan diseases. Toltrazuril, for example is used in treatment of coccidiosis interfering with intracellular developmental stages of parasites via effects on endoplasmic reticulum mitochondria and cell division, potentially via inhibition of succinate-cytochrome C reductase and NADH oxidase and succinate oxidase (Harder and Haberkorn, 1989).

Azithromycin is an antibiotic that binds a ribosomal subunit and is also effective in the prevention and treatment of cryptosporidiosis. The drug is carried to sites of infection, conveniently, by neutrophils and other myeloid cells (Parnham et al., 2014). Combination therapies are a way of improving outcomes and in the combined use of toltrazuril and azithromycine there is a notable synergistic effect on cryptosporidiosis in cows compared to either agent alone (Yagci et al., 2017).

## 5 Finding new therapeutic agents

**Figure 3:**
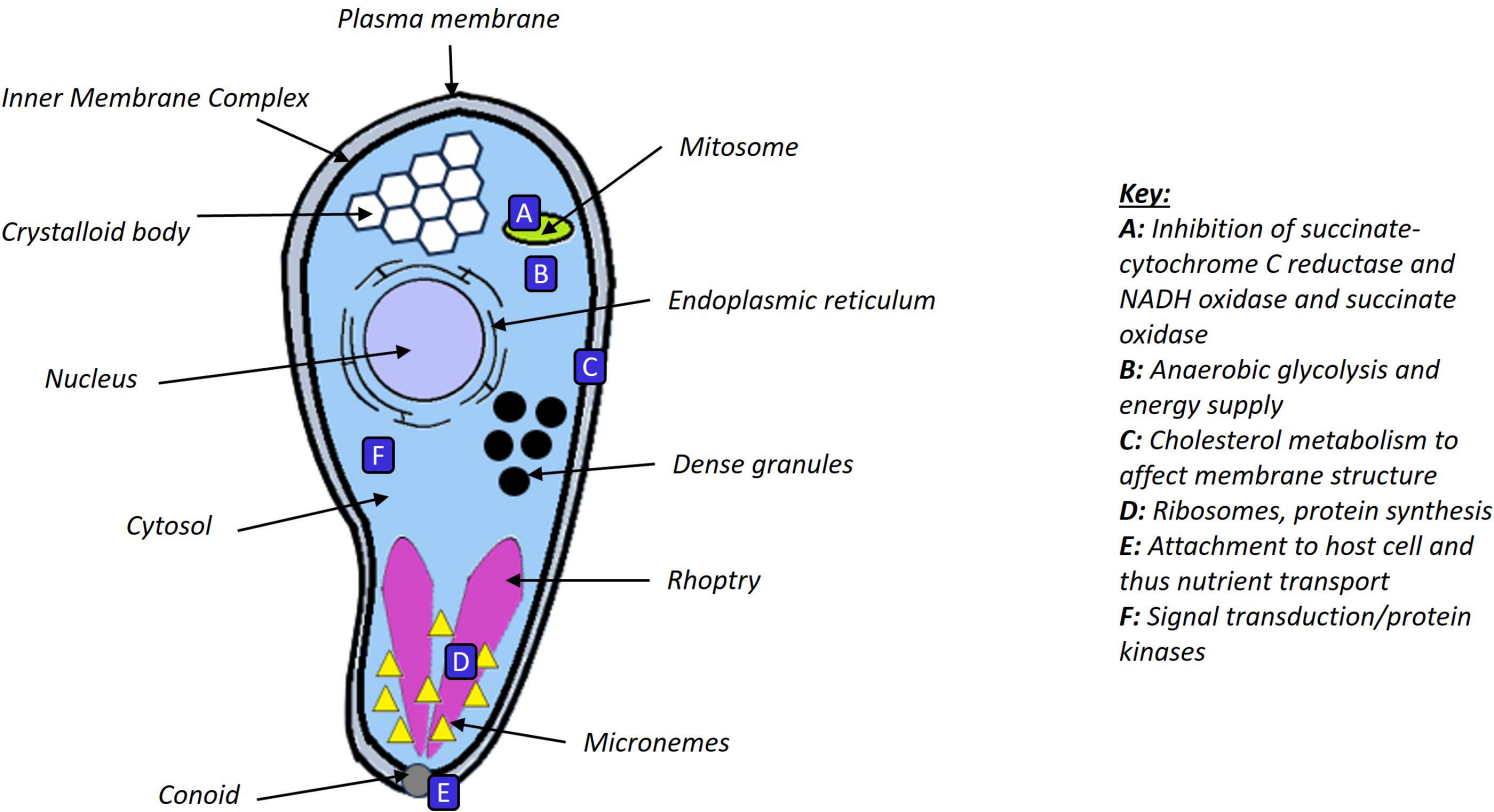
Potential drug target sites of *Cryptosporidium* sporozoites found in literature.

There are numerous classes of drug targets that are under investigation with respect to treatments in human disease. Given the enormous budgets put to these drug discovery and drug validation exercises, there is undoubted opportunity for learning and repurposing to treat diseases observed in livestock and companion animals. In respect of pharmacological intervention, several targets have been studied (**Figure 3**).

Prospectively the knowledge base on drug targets and putative inhibitory mechanisms in human disease will grow, and new classes of compounds will become available. Some of these may have application in veterinary medicine. A previous example would be the use of imatinib to treat malignant disease in dogs via inhibition of specific mutated, and thus activated, protein tyrosine kinases (Macedo et al., 2022). The compound was developed to treat Chronic Myeloid Leukaemia in humans and then developed for use in malignant disease in dogs. Below we take a longer view of the prospective areas where treatment for Cryptosporidiosis can possibly be found.

*Cryptosporidium* lacks most mitochondrial functions and cannot generate energy via oxidative phosphorylation (Mazurie et al., 2013). Biosynthesis capacity is relatively limited, thus, energy generation within the organism is restricted to anaerobic glycolysis and the Krebs cycle (Denton et al., 1996). This is not to stay that the organism cannot acquire amino acids and other biochemicals required for anabolic processes, from its host. Indeed, there is a major effect of the parasite on host cells inclusive of alteration in truncated microvilli, altered epithelial junctions and the formation of a complex host/pathogen interface which is clearly worthy of investigation for potential disruption. The parasite reliance on host biochemicals has been shown (Pawlowic et al., 2019), in this instance nucleic acids. There is *in vitro* evidence to support glycolysis as a drug target pathway (Vélez et al., 2021). Inhibitors of glycolytic enzyme hexokinase, and sugar transporters, lactate dehydrogenase inhibitors and glutamine metabolism inhibitors, reduce parasite replication. Inhibition of anaerobic glycolysis enzymes pyruvate kinase and lactate dehydrogenase both adversely affect *C.parvum* growth and survival in an *in vivo* model, but the combination of the two drugs has a synergistic effect. The effects on livestock animals and their microbiomes, however, may be so deleterious as to make the use of these drugs inappropriate (Khan et al., 2023).

In using the transcriptome data they have derived, Walzer et al. (2024) have given a deep insight into the various stages of the *Cryptosporidium* life cycle beyond morphological definition, and how these stages relate to patterns of gene expression. As has been seen in studies on embryological development, the use of such data will be of immense value to build a picture of life cycle stage and associated pathway activation (and thus opportunities for targeted therapy). *Cryptosporidium* has an elaborate interface with the host, including the feeder organelle (**Figures 1**, **2**), and there is clear evidence of a requirement for nutrients from the host. Transporter proteins as a target for drugs is an active area of research in human medicine, and as discussed above, the potential for targeted inhibition of substrate uptake into *Cryptosporidium* remains a possible way forward that would leave host physiology unaffected. Cryptosporidial infection has been shown to modulate intestinal epithelial glucose absorption and metabolism (Dengler et al., 2023) which is a vignette of how the parasitic organism alters biochemical flux pathways that can be targeted by drugs. For example, *C. parvum* invasion requires localised membrane insertion of Na^+^/glucose cotransporter 1 (SGLT1) using myosin aggregation (O'Hara et al., 2010), and SGLT1 inhibition decreases invasion (Chen et al., 2005). Membrane transporters are very much a subject of research for novel inhibitors to treat diseases. Biochemical transporters are increasingly being studied as drug targets (Galetin et al., 2024). For example, SGLT inhibitors (e.g., dapagliflozin, empagliflozin) have been approved as antihyperglycemic compounds for the treatment of type 2 diabetes (FDA, 2023). As such drugs become available in human medicine, they can then be considered for use in treatment for cryptosporidiosis when efficacy is clear, and the economic case can be made. Studies from human medicine more widely therefore offer new opportunities for inhibition, killing and removal of parasite load in the gut.

Microneme proteins of the parasite recognise carbohydrate moieties to anchor parasite to the host cell; this may be another area to target via disruption of the cell/parasite interaction. Lectins are relatively small molecules that bond carbohydrate moieties that are made by bacteria and plants. They have been considered for use in treatment of several diseases, including viral mediated diseases like HIV. Lectin delivery to the gut is feasible and as such, food supplements could have a prophylactic or treatment role in livestock (Lavín de Juan et al., 2017). However, lectins in feed can have adverse effects (Ansia and Drackley, 2020) thus a selective approach is clearly required. Nonetheless, lectins affect epithelial cells, by binding to the mucosa via a high affinity interaction with carbohydrates on cell surfaces (Friedman and Brandon, 2001). If specific lectins that targeted the GPI interaction between parasite and host were found, this may be beneficial for animal health in the face of parasitic ingestion. Lectins can be used as a delivery system and could therefore target antiparasitic drugs to parasites, however the economics of manufacture are a key issue as well as the development work required.

*Cryptosporidium* has the lowest number of paralogous proteins, meaning a specific drug targeting programme has more chance of successfully decreasing survival/proliferation of the parasite (Mazurie et al., 2013). Extensive review of organelle proteomics in *Cryptosporidium* has revealed fascinating detail on the constituents of rhoptry, oocyst wall, secreted granules, microneme and plasma membrane. This key analysis identifies proteins with extracellular domains that can be considered drug targets (Guérin et al., 2023). These proteins reorder the epithelium and thus are key to the parasitic life cycle. An example of one area worthy of further investigation is the fact that the oocyst wall proteome includes patched family proteins that regulate lipid transport. This lipid transport helps to build the structure that makes the oocyst resistant to chemical attack or disinfectant (Samuelson et al., 2013). A patched related protein in plasmodium is key in membrane integrity of *Plasmodium falciparum* and its inhibition makes this organism more susceptible to a detergent like compound (Istvan et al., 2019). Related proteins are inhibited by specific small molecule drugs but compounds that can inhibit these processes have never been contextualised into cryptosporidial antiparasitic action (Wu et al., 2022). However, in a malarial model, U18666A drug, that leads to cholesterol sequestration, leads to restriction of parasite growth likely via said cholesterol sequestration (Petersen et al., 2017). Another cholesterol-lowering drug employed in human medicine, ezetimide, blocks *Toxoplasma. gondii, B. besnoiti* and *N. caninum* tachyzoite infectivity and replication in primary bovine endothelial host cells. Perhaps just as important as the inhibitory effects is the concept that the drug yields a more disinfectant sensitive population of oocysts (Larrazabal et al., 2021b). High-density lipoprotein (a cholesterol carrying protein in the blood) receptor SR-BI also reduced invasive capacities of the above apicomplexans (Larrazabal et al., 2021a). In other words, there is growing evidence that the modulation of cholesterol metabolism using several different drugs in anticomplexan-related diseases could have prophylactic, or disease treatment value. As the cholesterol to phospholipid ratio in plasma membranes is about 0.7 the importance of this steroid is clear. Specifically, cholesterol content reduction can alter membrane permeability and signal transduction (Blok et al., 1977; Whetton et al., 1983; Shaheen et al., 2023).

Many different studies have revealed that a large number of apicomplexan specific proteins are secreted by *Cryptosporidium* or are associated with the parasite/host interface. These have been detailed in a comprehensive and elegant review (Plattner and Soldati-Favre, 2008). These studies do suggest opportunities for disruption of rhoptry/microneme parasite protein interactions as a way forward for intervention in cryptosporidiosis infection. *Cryptosporidium* is epiparasitic, with a peripheral cellular interaction, where only a part of the parasite is embedded in the host cell. As knowledge of the unique proteins found in the cellular interface develops (Guérin et al., 2023), new means of targeting the parasite at an accessible site (the interface) will become apparent. Protein-protein interactions (PPIs) are now regarded as druggable targets (Bojadzic and Buchwald, 2018; Zhu et al., 2022). This includes cell surface signalling complexes such as CD40-CD40 ligand. Given the primary structure (amino acid sequence) of (*Cryptosporidium* specific) parasite proteins, the tertiary structure (folded shape) can be predicted using models that predict the shape of proteins, such as Alphafold (Jumper et al., 2021). In the future this will enable the Computer Aided Design (Hurwitz et al., 2022) of peptide drugs that specifically bind to key functional parasitic proteins (Wang et al., 2022).This will be after standard biochemical, plus computer-based approaches in proteomics, have identified membrane bound *Cryptosporidium* proteins to membrane bound host protein interactions (Ren et al., 2022). As stated, *Cryptosporidium* has a low number of paralogous proteins meaning a specific drug effect is more probable. Such drug targets may best be for the epiparasitic pathogen/host interactions. Cost of manufacture, specificity and efficacy need to be considered in these future avenues for research. Small molecule inhibitors are often too small to inhibit PPIs. A contact area of 1500–3000 A^2^ usually defines a PPI, but small molecules only cover 300–1000 A^2^ of the protein surface. Peptide drugs (and modified peptide drugs) have a greater size and thus, with specific design, can act as potent inhibitors of PPIs. The clinical use of PPIs offers a higher specificity than many low molecular weight inhibitors (Wang et al., 2022). Furthermore, peptides are already widely used in veterinary medicine.

A different approach to cryptosporidiosis is screening for drug repurposing. This can play a major role in finding economically viable treatments for cryptosporidiosis, in part because first human trials have already been performed and possibly phase 2/3 clinical trials. Plainly, repurposing for use in livestock wellness would require further work but would be markedly cheaper than starting with no information on molecular structure, pharmacodynamics/kinetics and on *in vivo* effects. A high-content imaging assay for inhibitors of *C.parvum* proliferation within a human intestinal epithelial cell line has been used to find prospective new treatments. This approach screened 78,942 known compounds and found 12 drugs with activity against *Cryptosporidium*. These included an inhibitor of Protein kinase C α and β, and the JAK2 tyrosine kinase. In respect of JAK2 kinase inhibitors, these have already been deployed as treatment of human disease (e.g. polycythaemia vera, myelofibrosis) and for veterinary medicine in companion animals (Shawky et al., 2022). Protein kinase C inhibitors have been thoroughly investigated and it remains to be seen if they have a substantial clinical impact. Given the wealth of data on JAK2 inhibitors and the fact that one such inhibitor, Ruxolitinib (targets *JAK1/2)* has a European Union patent that is expected to expire in August 2027 and the US patent is expected to expire in December 2027 offers opportunity for use in parasitic disease treatment. JAK2 also has a role in mastitis and milk production in cows (Khan et al., 2020) providing a possible use elsewhere in dairy farming. Bumped kinase inhibitor has shown promise as an inhibitor of *Cryptosporidium* growth, likely via calcium-dependent protein kinase 1 (CDPK1) inhibition (Castellanos-Gonzalez et al., 2013). Novel artificial intelligence-based assessment of kinase structure and inhibitor binding will in the future enable increased speed of development for kinase inhibitors in veterinary and human medicine (Liu et al., 2023). Screens for inhibition of inosine-5’-monophosphate dehydrogenase have also shown some promise (Gorla et al., 2014) as have polyamine analogs, but there is no evidence of clinical development of these drugs (Yarlett et al., 2007).

The major finding of this above drug repurposing screening was the effect of clofazimine, an antibiotic used in treatment of leprosy, on *Cryptosporidium*. The mechanism of action of clofazimine is not known but it is believed to be related to effects on membrane structures or membrane-bound proteins. Whilst clofazimine has limited oral bioavailability, it has been encapsulated to promote higher absorption in the treatment of leprosy. The intestinal permeability and hydrophobic nature of clofazimine enables the drug to be retained in the gut, where *Cryptosporidium* is found (Baik et al., 2013).

Recently, analysis of the *Cryptosporidium* transcriptome has been performed at the single cell level. This extensive analysis of the parasite at different developmental stages has much to offer in respect of defining specific molecular targets to induce death in the parasite. As one example, the Myb-M gene has been shown to be key in sex determination in the parasite (Walzer et al., 2024). In *Cryptosporidium*, sex is key in respect of infection and transmission. Thus, Myb-M could be a target to adversely affect parasite replication. In humans Myb is a transcription factor associated with haematopoiesis and leukemogenesis (Pattabiraman et al., 2014; Wang et al., 2018). Thus, it has been viewed as a drug target, even though transcription factors are considered difficult targets against which to develop small molecule inhibitors. The human Myb interaction with p300 protein by which it elicits its effects has however been the subject of interest and is therefore an attractive target for prevention and intervention. Plumbagin and naphthoquinones have been shown to inhibit Myb/p300 interaction (Uttarkar et al., 2016; Nicosia et al., 2023). Thus, consideration of Myb-M structure using protein structure prediction (Bouatta and AlQuraishi, 2023) and consideration of these new molecules and others as protein:protein interaction inhibitors (Zhang et al., 2024) may offer opportunities for treatment/prevention of cryptosporidiosis.

There are currently no vaccines available to treat cryptosporidiosis (Innes et al., 2020). This is due to the disease largely affecting neonatal animals which would have difficulty rapidly generating protective immunity through active vaccination. Recent preliminary studies have investigated experimental vaccines for dam or calf vaccination protocols with promising results, though these were shown to be unsuccessful in large field trials; therefore, they are not currently marketed (Jenkins, 2004; Maier et al., 2022). Consequently, interventions address prevention, control, and supportive therapies post-infection. Potentially mRNA vaccines may be of benefit in the future as novel targets emerge and cost per vaccination decreases (Savinkina et al., 2022).

The adoption of a One Health approach offers more opportunities to control the spread of cryptosporidiosis, particularly from zoonotic species such as *C. parvum* (Innes et al., 2020). Investigation and control of Cryptosporidium in production animals ultimately leads to lower zoonotic risk and better public health. Development of disease surveillance systems to identify the source of infection and route of transmission in both humans and animals is essential to controlling outbreaks (Hotchkiss et al., 2015). For example, emerging technologies for early detection of waterborne pathogens are enabling improved water and public safety (Innes et al., 2020), though there is still a way to go. Another element of the One Health approach is to take the learning from drug development strategies in human disease and apply them assiduously and with consideration of health-economic issues to parasitology and veterinary medicine.

## Author contributions

HR: Visualization, Writing – original draft, Writing – review & editing. AC: Writing – review & editing. AW: Conceptualization, Supervision, Writing – review & editing.
