# Supplementary material for: Understanding the Cryptosporidium species and their challenges to animal health and livestock species for informed development of new, specific treatment strategies
Source: Front Parasitol. 2024 Aug 6;3:1448076. doi: 10.3389/fpara.2024.1448076 (PMC11732131; doi:10.3389/fpara.2024.1448076)
Supplement: Supplementary file 1 [file Table_1.docx]

Understanding the *Cryptosporidium* species and their challenges to animal health and livestock species for informed development of new, specific treatment strategies

Supplementary Material

# Appendix 1:

Table 3. The variation of reported *Cryptosporidium* prevalence per region, per species.

| **Species** | **Region** | **Prevalence (%)** | **Reference** |
| --- | --- | --- | --- |
| Cattle | Europe | 13.8 | (Imre and Darabus, 2011) |
|  | US | 20.5 | (Fayer, Santín and Dargatz, 2010) |
|  | China | 18.8 | (Chen and Huang, 2012) |
|  | Kuwait | 33.1 | (Majeed *et al.,* 2022) |
|  | Egypt | 18.8 | (Gattan *et al.,* 2023) |
|  | Ethiopia | 13.8 | (Ebiyo and Haile, 2022) |
|  | South America | 18 | (Nakashima *et al.,* 2022) |
| Pigs | Global | 16.3 | (Chen, Y. *et al.,* 2023). |
|  | Asia | 55.8 | (Helmy, Y. and Hafez, 2022) |
| Sheep | Global | 18.9 | (Checkley *et al.,* 2015) |
|  | Asia | 14.8 |  |
|  | Europe | 20 |  |
|  | Africa | 21.7 |  |
|  | North America | 29.3 |  |
|  | South America | 20.3 |  |
|  | Oceania | 19.1 |  |
| Chickens | US | 41 | (de Graaf *et al.,* 1999) |
|  | Asia | 10 | (Helmy *et al.,* 2017) |
|  | Europe | 7 | (Wang *et al.,* 2014) |
|  | South America | 14.8 | (da Cunha, Cury and Santín, 2018) |
|  | North Africa | 11.9 | (Olonisakin and Olusi, 2023). |

# References:

Checkley, W., White, A.C., Jaganath, D., Arrowood, M.J., Chalmers, R.M., Chen, X., Fayer, R., Griffiths, J.K., Guerrant, R.L., Hedstrom, L., Huston, C.D., Kotloff, K.L., Kang, G., Mead, J.R., Miller, M., Petri, W.A., Priest, J.W., Roos, D.S., Striepen, B., Thompson, R.C.A., Ward, H.D., Van Voorhis, W.A., Xiao, L., Zhu, G. and Houpt, E.R. (2015) 'A review of the global burden, novel diagnostics, therapeutics, and vaccine targets for cryptosporidium', *The Lancet Infectious Diseases,*15(1), pp. 85-94 Available at: 10.1016/S1473-3099(14)70772-8.

Chen and Huang (2012) 'Prevalence and molecular characterization of Cryptosporidium spp. in dairy cattle from farms in China', *Journal of Veterinary Science,*13(1), pp. 15-22 Available at: 10.4142/jvs.2012.13.1.15.

Chen, Y., Qin, H., Wu, Y., Xu, H., Huang, J., Li, J. and Zhang, L. (2023) 'Global prevalence of Cryptosporidium spp. in pigs: a systematic review and meta-analysis', *Parasitology,*150(6), pp. 531-544 Available at: 10.1017/S0031182023000276.

da Cunha, M.J.R., Cury, M.C. and Santín, M. (2018) 'Molecular characterization of Cryptosporidium spp. in poultry from Brazil', *Research in Veterinary Science,*118, pp. 331-335 Available at: 10.1016/j.rvsc.2018.03.010.

de Graaf, D.C., Vanopdenbosch, E., Ortega-Mora, L.M., Abbassi, H. and Peeters, J.E. (1999) 'A review of the importance of cryptosporidiosis in farm animals', *International Journal for Parasitology,*29(8), pp. 1269-1287 Available at: 10.1016/S0020-7519(99)00076-4.

Ebiyo, A. and Haile, G. (2022) 'Prevalence and Factors Associated with Cryptosporidium Infection in Calves in and around Nekemte Town, East Wollega Zone of Ethiopia', *Veterinary Medicine International,*2022, pp. 1468242 Available at: 10.1155/2022/1468242.

Fayer, R., Santín, M. and Dargatz, D. (2010) 'Species of Cryptosporidium detected in weaned cattle on cow–calf operations in the United States', *Veterinary parasitology,*170(3), pp. 187-192 Available at: 10.1016/j.vetpar.2010.02.040.

Gattan, Alshammari, Marzok, Salem, AL-Jabr and Selim (2023) 'Prevalence of Cryptosporidium infection and associated risk factors in calves in Egypt', *Scientific Reports,*13(1), pp. 1-9 Available at: 10.1038/s41598-023-44434-7.

Helmy, Krücken, Abdelwhab, Samson-Himmelstjerna and Hafez (2017) 'Molecular diagnosis and characterization of Cryptosporidium spp. in turkeys and chickens in Germany reveals evidence for previously undetected parasite species', *PLOS ONE,*12(6), pp. e0177150 Available at: 10.1371/journal.pone.0177150.

Helmy, Y. and Hafez, H. (2022) 'Cryptosporidiosis: From Prevention to Treatment, a Narrative Review', *Microorganisms,*10(12), pp. 2456 Available at: <https://doi.org/10.3390/microorganisms10122456.>

Imre, K. and Darabus, G. (2011) 'Distribution of Cryptosporidium species, genotypes and C. parvum
subtypes in cattle in European countries', *Science of Parasitology,*12(1).

Majeed, Q.A.H., AlAzemi, M.S., Al-Sayegh, M.T. and Abdou, N.M.I. (2022) 'Epidemiological and Molecular Study of Cryptosporidium in Preweaned Calves in Kuwait', *Animals : an Open Access Journal from MDPI,*12(14), pp. 1805 Available at: 10.3390/ani12141805.

Nakashima, F.T., Fonseca, A.B.M., Coelho, L.F.d.O., Barbosa, A.d.S., Bastos, O.M.P. and Uchôa, C.M.A. (2022) 'Cryptosporidium species in non-human animal species in Latin America: Systematic review and meta-analysis', *Veterinary Parasitology: Regional Studies and Reports,*29, pp. 100690 Available at: 10.1016/j.vprsr.2022.100690.

Olonisakin, A.A. and Olusi, T.A. (2023) 'Epidemiology of Cryptosporidium sp. infection among free-range and intensive farm birds in Akure South LGA, Ondo State, Nigeria | Bulletin of the National Research Centre | Full Text', *Bulletin of the National Research Centre,*45(174) Available at: <https://doi.org/10.1186/s42269-021-00632-9.>

Wang, Y., Yang, W., Cama, V., Wang, L., Cabrera, L., Ortega, Y., Bern, C., Feng, Y., Gilman, R. and Xiao, L. (2014) 'Population genetics of Cryptosporidium meleagridis in humans and birds: evidence for cross-species transmission', *International journal for parasitology,*44(8), pp. 515-521 Available at: 10.1016/j.ijpara.2014.03.003.
